# The Extracellular Lipopeptides and Volatile Organic Compounds of *Bacillus subtilis* DHA41 Display Broad-Spectrum Antifungal Activity against Soil-Borne Phytopathogenic Fungi

**DOI:** 10.3390/jof9080797

**Published:** 2023-07-28

**Authors:** Dhabyan Mutar Kareem Al-Mutar, Muhammad Noman, Noor Salih Abduljaleel Alzawar, Hadi Hussein Qasim, Dayong Li, Fengming Song

**Affiliations:** 1 Key Laboratory of Crop Diseases and Insect Pests of Ministry of Agriculture, Institute of Biotechnology, Zhejiang University, Hangzhou 310058, China; dhabyan@zju.edu.cn (D.M.K.A.-M.); m.noman@zju.edu.cn (M.N.); dyli@zju.edu.cn (D.L.); 2 Key Laboratory of Biology of Crop Pathogens and Insects of Zhejiang Province, Institute of Biotechnology, Zhejiang University, Hangzhou 310058, China; 3 Basra Agriculture Directorate, Almudaina 61008, Iraq; hadialzayadi67@gmail.com; 4 Ministry of Agriculture, Directorate of Agriculture Extension and Training, Albasra 61001, Iraq; masterbiology85@gmail.com

**Keywords:** antifungal activity, *Fusarium oxysporum* f. sp. *niveum*, lipopeptide, soil-borne pathogens, volatile organic compounds

## Abstract

*Fusarium oxysporum* f. sp. *niveum* (*Fon*) is a devastating soil-borne fungus causing Fusarium wilt in watermelon. The present study investigated the biochemical mechanism underlying the antifungal activity exhibited by the antagonistic bacterial strain DHA41, particularly against *Fon*. Molecular characterization based on the *16S rRNA* gene confirmed that DHA41 is a strain of *Bacillus subtilis*, capable of synthesizing antifungal lipopeptides, such as iturins and fengycins, which was further confirmed by detecting corresponding lipopeptide biosynthesis genes, namely *ItuB*, *ItuD*, and *FenD*. The cell-free culture filtrate and extracellular lipopeptide extract of *B. subtilis* DHA41 demonstrated significant inhibitory effects on the mycelial growth of *Fon*, *Didymella bryoniae*, *Sclerotinia sclerotiorum*, *Fusarium graminearum*, and *Rhizoctonia solani*. The lipopeptide extract showed emulsification activity and inhibited *Fon* mycelial growth by 86.4% at 100 µg/mL. Transmission electron microscope observations confirmed that the lipopeptide extract disrupted *Fon* cellular integrity. Furthermore, *B. subtilis* DHA41 emitted volatile organic compounds (VOCs) that exhibited antifungal activity against *Fon*, *D. bryoniae*, *S. sclerotiorum*, and *F. graminearum*. These findings provide evidence that *B. subtilis* DHA41 possesses broad-spectrum antifungal activity against different fungi pathogens, including *Fon*, through the production of extracellular lipopeptides and VOCs.

## 1. Introduction

Soil-borne fungi belonging to the *Fusarium*, *Rhizoctonia*, *Sclerotinia*, and *Verticillium* genera can cause root rot, vascular wilt, damping-off, and sclerotinia stem rot diseases in economically important crops, including vegetables, rice, wheat, cotton, and fruits [1]. Among them, the *Fusarium oxysporum* species complex can infect more than 150 crop species, including watermelon, tomato, melon, and cotton, and cause severe vascular Fusarium wilt diseases, leading to serious yield loss worldwide [2]. For example, *F. oxysporum* f. sp. *niveum* (*Fon*) causes the most destructive vascular wilt in watermelon, resulting in 30–50% yield losses [3]. Chemical pesticides have become ineffective due to their narrow spectrum activity and non-target environmental impacts, rendering them less effective in disease control. Furthermore, soil-borne fungi can thrive and persist by forming resting structures for extended periods [1], posing challenges to infection prevention. Therefore, soil-borne phytopathogenic fungi have long been a serious threat to sustainable agricultural production, emphasizing the development of novel eco-friendly green strategies to manage these fungal diseases in crops effectively.

Biological control of soil-borne plant diseases using beneficial microbes and their active metabolites is an effective and sustainable alternative to chemical pesticides [4,5]. Among these beneficial microbes, bacterial strains from the genus *Bacillus*, such as *Bacillus subtilis*, *Bacillus velezensis*, and *Bacillus amyloliquefaciens*, have been extensively exploited for their potentials in managing soil-borne fungal diseases [6,7,8,9]. Different *Bacillus* species (spp.) have been shown to possess significant antagonistic activity against soil-borne pathogenic fungi, including *Fusarium* spp., *S. sclerotiorum*, and *R. solani* [10,11,12,13,14,15,16]. Importantly, numerous *Bacillus* spp. have shown great potential in controlling Fusarium wilt in different crops [17]. Notably, *B. subtilis*, *B. velezensis*, *Bacillus tequilensis*, and *Bacillus licheniformis* have demonstrated the ability to protect watermelon, cucumber, tomato, flax, and banana from Fusarium wilt [12,16,18,19,20,21]. For example, *B. velezensis* F21 significantly suppressed watermelon Fusarium wilt, reducing the disease incidence in the greenhouse (80%) and in the field (66%) [19], resulting in improved crop yield and quality in an eco-friendly and cost-effective manner. Similarly, *B. amyloliquefaciens* DHA55 protected watermelon plants against Fusarium wilt under greenhouse conditions, suppressing disease incidence up to 75% [16]. These facts highlight the promising biocontrol potential of *Bacillus* spp. against devastating soil-borne diseases in crops; however, their underlying action mechanisms need to be dissected.

*Bacillus* spp. employ various direct and indirect mechanisms to protect plants against soil-borne pathogens, including the secretion of hydrolytic enzymes (e.g., chitinase, protease, and β-1,3-gucanse), the production of antimicrobial metabolites, and the priming of plant-induced systemic resistance (ISR) [7,17]. *B. subtilis* and *B. licheniformis* secrete extracellular chitinase and protease against *F. oxysporum*, inhibiting mycelial growth [13,22,23]. *B. cereus*, *B. subtilis*, and *B. fortis* were found to activate ISR against Fusarium wilt in tomato and banana through their regulation of jasmonic acid-, ethylene-, and brassinosteroid-mediated defense signaling pathways [24,25,26,27,28]. However, the production of extracellular antifungal metabolites by *Bacillus* spp., particularly non-ribosomally synthesized lipopeptides and volatile organic compounds (VOCs), is the primary disease-suppressive mechanism that directly inhibits soil-borne pathogens [29,30,31,32,33,34,35]. Extracellular lipopeptides, including iturins, fengycins, bacillomycin, and surfactins, are widely distributed in *Bacillus* spp. [29,31]. Iturin A and fengycins from *B. amyloliquefaciens* and *B. velezensis* have been shown to significantly inhibit the mycelial growth of *F. oxysporum* f. sp. *niveum* (*Fon*), *F. oxysporum* f. sp. *lycopersici*, *R. solani*, and *S. sclerotiorum* [16,36,37,38,39]. Furthermore, *B. amyloliquefaciens* and *Bacillus mycoides* BM02 have been discovered to emit VOCs to inhibit the mycelial growth and spore germination of *F. oxysporum* and *S. sclerotiorum* [40,41,42,43]. These extracellular lipopeptides and VOCs produced by *Bacillus* spp. hold potential as biopesticides for managing soil-borne crop diseases [31,33].

Fusarium wilt, caused by *Fon*, poses a serious threat to the watermelon industry, particularly in the greenhouse monocropping system [44,45]. We previously initiated an effort to develop novel sustainable biocontrol strategies for managing watermelon Fusarium wilt and characterized six antagonistic bacterial strains, including DHA41, that demonstrated strong antifungal activity against different phytopathogenic fungi, including *Fon*, and effectively suppressed watermelon Fusarium wilt in greenhouse experiments [16]. In this study, we investigated the biochemical basis underlying the antifungal activity of strain DHA41. Our findings suggest that the DHA41 strain produces three families of lipopeptides and emits VOCs, enabling it to exhibit antifungal activity against soil-borne pathogenic fungi such as *Fon*, *Didymella bryoniae*, *R. solani*, *Fusarium graminearum*, and *S. sclerotiorum*.

## 2. Materials and Methods

### 2.1. Growth Conditions for Bacterial Strain DHA41 and Fungal Pathogens

Bacterial strain DHA41 was isolated from watermelon rhizosphere soil using the serial dilution method and cultured on a Luria–Bertani (LB) plate at 28 ± 2 °C, as described previously [16]. Fungal pathogens, including *F. oxysporum* f. sp. *Niveum* (*Fon*), *F. graminearum* (*Fg*), *D. bryoniae* (*Db*), *R. solani* (*Rs*), and *S. sclerotiorum* (*Ss*), were collected from the Crop Diseases and Insect Pests Laboratory of MARA at Zhejiang University and maintained on potato dextrose agar (PDA) at 28 ± 2 °C [16]. For sporulation, *Fon* was cultured in a mung bean liquid medium at 28 ± 2 °C with shaking (150 rpm) for 2 d, and spores were collected and adjusted to a final concentration of 4 × 10^6^ spores/mL [16].

### 2.2. Amplification of 16S rRNA and Lipopeptide Biosynthesis Genes

Bacterial strain DHA41 was cultured in liquid LB medium under shaking (180 rpm) at 28 ± 2 °C for 12 h. The bacterial cells were collected by centrifugation, and genomic DNA was extracted using the Takara MiniBEST Bacteria Genomic DNA Extraction Kit Ver.3.0 (Takara, Dalian, China), following the given instructions. Fragments of the *16S rRNA* and the lipopeptide biosynthesis genes, namely *ItuB*, *ItuD*, and *FenD*, were amplified using gene-specific primers (Table 1). The *16S rRNA* gene amplicon was sequenced commercially (Zhejiang YouKang Biotech, Hangzhou, China), aligned using the ClustalX program [46], and subjected to phylogenetic analysis using MEGA 11.0 software, following the neighbor joining (NJ) method [47].

### 2.3. Characterization of Cellular Fatty Acids

Bacterial strain DHA41 was cultivated on Tryptic soy agar (Difco Laboratories, Sparks, MD, USA) at 28 ± 2 °C for 24 h. Fatty acid methyl esters (FAMEs) were prepared and analyzed following the protocol of the Sherlock microbial identification system [50]. Briefly, fatty acids were released from the bacterial cells through saponification with NaOH and esterified with 6 N HCl to generate FAMEs. The FAME-containing upper layer was then collected using a methyl tert-butyl ether and hexane solution (1:1, *v*/*v*). The FAME profiling was conducted using a Hewlett Packard 5890 Series gas chromatography machine (Ramsey, MN, USA). The fatty acids were identified and quantified by comparing the retention time and peak area with an authentic standard fatty acid mixture (Sigma-Aldrich, St. Louis, MO, USA) as well as with the RTSBA6 6.10 library of bacterial fatty acids [50].

### 2.4. Extraction, Purification, and Characterization of Extracellular Lipopeptides from DHA41

Bacterial strain DHA41 was grown in 100 mL liquid LB medium at 28 ± 2 °C with shaking (200 rpm) for 72 h. The culture was centrifuged (12,000 rpm) at 4 °C for 20 min, and the resultant supernatant was collected. The supernatant was acidified by adding 2 M HCl (pH 2.0) and incubated overnight at 4 °C. Lipopeptide precipitates were collected through centrifugation (15,000 rpm) and resuspended in a methanol and water solution (2:1, *v*/*v*). The lipopeptide extract was dried in a rotary vacuum at 40 °C, re-dissolved in dimethyl sulfoxide (DMSO), and stored at −20 °C for further investigation.

Matrix-assisted laser desorption/ionization–time-of-flight mass spectrometry (MALD-TOF-MS) analysis was used to identify the lipopeptides of strain DHA41 [51]. A single colony of bacterial strain DHA41 was picked and homogenized in a matrix solution, as described previously [16]. After centrifugation (12,000 rpm) for 20 min at 4 °C, 1 μL of the resulting supernatant was spotted onto a MALDI-TOF-MS target plate (Bruker Daltonik, Bremen, Germany) and air-dried. The samples were analyzed using an Ultraflex MALDI-TOF-MS spectrometer equipped with a smartbeam laser (Daltonics, Bremen, Germany) for desorption and ionization with a nitrogen laser at 337 nm. The obtained spectra were analyzed to identify different lipopeptides in the extract, with the molecular weight ranging from 800 to 3000 Daltons (Da).

### 2.5. Evaluation of Emulsification Index

The emulsification index was evaluated following a previous protocol [52]. Briefly, bacterial strain DHA41 was grown in 100 mL liquid LB medium at 28 ± 2 °C with shaking (200 rpm) for 72 h. A cell-free supernatant (2 mL) was obtained after centrifugation (12,000 rpm) and mixed with 3 mL of hydrophobic compounds (sunflower oil, mineral oil, and toluene) or a sodium dodecyl sulfate (0.1 g/L; Sigma-Aldrich, St. Louis, MO, USA) and Triton X-100 solutions (0.1%, *v*/*v*; Sigma-Aldrich, St. Louis, MO, USA) as controls. The mixture was thoroughly vortexed for 2 min and left at room temperature for 24 h. The height of the stable emulsion layer was measured, and the emulsification index (%) was calculated by comparing the height of the emulsified layer to the total height of the liquid.

### 2.6. Antifungal Activity of Cell-Free Supernatant, Lipopeptide Extract, and VOCs of DHA41

The antifungal activity of the cell-free supernatant of strain DHA41 and its lipopeptide extract was examined using the well culture method [53,54], with minor modifications. The filter-sterilized cell-free supernatant (30 µL), obtained from a 2-day-old culture of strain DHA41, and 30 µg/mL of lipopeptide extract (60 µL) were poured into the wells (5 mm) of the PDA plates, which were then inoculated with fungal mycelial plugs (5 mm in diameter). The wells supplemented with a similar volume of sterile LB medium or DMSO were used as controls, followed by incubations at 28 ± 2 °C for 5 d. To estimate growth inhibition rate, the colony diameters grown in the treated plates were compared to those grown in the control plates. 

The effect of VOCs produced by DHA41 on the tested fungi was also examined using the two-sealed-base-plates method as previously described [55]. Briefly, bacterial strain DHA41 was streaked onto the LB medium on one base plate, while fungal mycelial discs (5 mm in diameter) were placed on PDA on another base plate. The two plates were then tightly assembled face-to-face and sealed with three layers of Parafilm and incubated at 28 ± 2 °C for 3–5 d. Two-plate assemblies without inoculation of strain DHA41 served as controls. The growth inhibition rate was calculated by comparing the colony diameters grown in the presence of strain DHA41 to those grown on the control plates.

### 2.7. Minimal Inhibitory Concentration Assay 

The minimal inhibitory concentration (MIC) of the lipopeptide extract against *Fon* was determined as previously described [16]. Briefly, *Fon* inoculum (50 µL) was added to the wells of a 96-well microtiter plate, followed by supplementation with varying levels of lipopeptide extract (50, 75, 100, 200, and 300 µg/mL). Control wells were supplied with DMSO. After incubation at 28 ± 2°C for 24 h, the optical density at 600 nm (OD_600_) was measured. To calculate the growth inhibition rate, the OD_600_ values in the lipopeptide-supplemented wells were compared to that in the control wells.

### 2.8. Microscopic Observation

The effect of lipopeptide extract on *Fon* cell viability was examined by staining using fluorescein diacetate (FDA) and propidium iodide (PI), as previously described [56,57]. In these experiments, live fungal cells exhibited green fluorescence, while dead cells showed red fluorescence. After treating the *Fon* inoculum with lipopeptide extract (30 µg/mL) for 12 h, fungal mycelia were collected and resuspended in DMSO, followed by staining with fluorescent dyes (FDA and PI) at room temperature for 15 min under dark conditions. The stained mycelia were observed with a Zeiss LSM 880 laser confocal microscope (Jena, Germany).

The effect of the lipopeptide extract on *Fon* mycelial and conidial cellular structure was examined using transmission electron microscopy (TEM, model H-7650, Hitachi, Tokyo, Japan), as previously described [57,58]. The *Fon* inoculum (1 mL) was treated with lipopeptide extract (30 µg/mL) and incubated with shaking (150 rpm) at 28 ± 2 °C for 12 h. The samples were then immersed in 2.5% glutaraldehyde overnight, rinsed three times for 15 min with 0.1 M phosphate buffer (pH 7.0), and post-fixed in OsO_4_ in the phosphate buffer (1%, *w*/*v*) for 2 h. The samples were dehydrated in an ethanol grade (30–70%) for 15 min at each concentration and finally dehydrated twice in absolute acetone for 20 min. The dehydrated samples were embedded in Spurr resin and polymerized for 12 h at 70 °C. Ultrathin sections of the samples were stained with uranyl acetate and lead citrate and observed using the H-7650 TEM. 

### 2.9. Statistical Analysis

The experiments in this study were independently conducted three times, and at least three replicates were included for each treatment in an independent experiment. Data from three independent experiments were subjected to statistical analysis using the Tukey’s least significant difference (LSD) method to determine the statistical significance among the treatments at a 95% confidence level in SPSS 14.0 software.

## 3. Results

### 3.1. Molecular and Biochemical Characterization of B. subtilis DHA41

**Figure 1:**
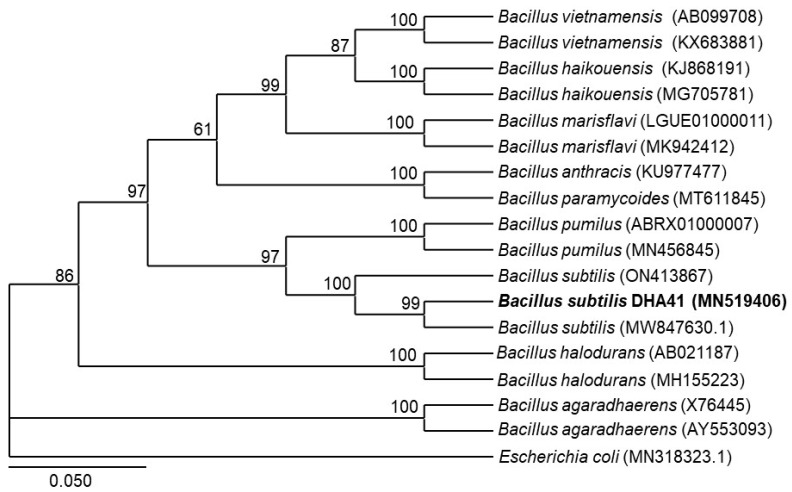
Molecular characterization of the antagonistic bacterial strain DHA41. The phylogenetic tree of strain DHA41’s *16S rRNA* gene (bold text), with those from other *Bacillus* species, was constructed using the neighbor joining method with a 1000-bootstrap approach. The *Escherichia coli* 16S *rRNA* gene sequence (MN318323.1) was used as an outgroup in the tree. The scale bar indicates that the sequences represented in the tree differ by an average of 0.050 nucleotide substitutions per site.

To characterize strain DHA41, a 1081 bp fragment of the *16S rRNA* gene was amplified and sequenced. In the phylogenetic tree, the *16S rRNA* gene sequence was closely grouped with *B. subtilis* (ON413867 and MW847630.1) and showed >97% similarity to the *16S rRNA* genes of these closely-related groups (Figure 1). For biochemical characterization, the fatty acid composition of strain DHA41 was analyzed, revealing 16:0 (15.42%), 15:0 iso (24.55%), 15:0 anteso (34.10%), 17:0 iso (6.65%), and 17:0 anteso (5.20%) as the most abundant saturated fatty acids (Table 2). This fatty acid composition is similar to those in reported *Bacillus* spp. [59,60], confirming that the DHA41 strain belongs to the genus *Bacillus*. Overall, the molecular phylogenetic relationship suggests the taxonomic identity of the bacterial strain DHA41 as *Bacillus subtilis*.

### 3.2. Identification of Extracellular Lipopeptides Produced by B. subtilis DHA41

**Figure 2:**
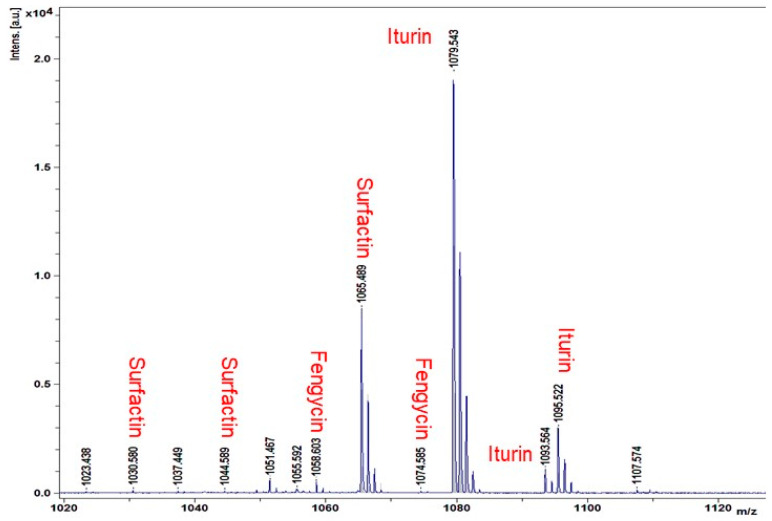
Identification of extracellular lipopeptides produced by *B. subtilis* DHA41 by matrix-assisted laser desorption/ionization–time-of-flight mass spectrometry.

**Figure 3:**
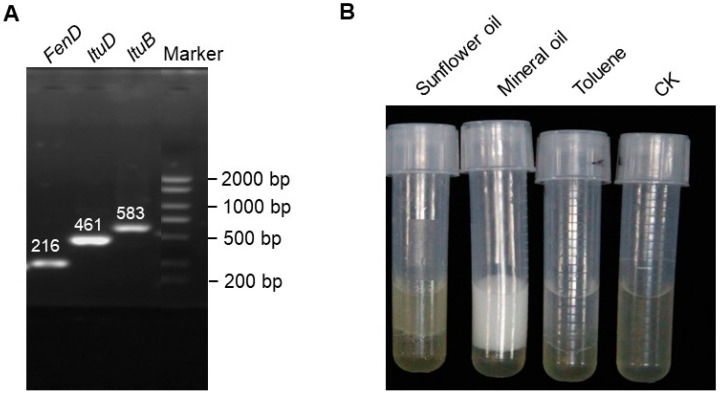
Detection of the lipopeptide biosynthesis genes in *B. subtilis* DHA41 (**A**) and analysis of the emulsification index of *B. subtilis* DHA41 on different organic oil substrates (**B**). The amplicon sizes are indicated above the bands in (**A**).

The extracellular lipopeptides produced by *B. subtilis* DHA41 were characterized by MALDI-TOF-MS analysis, showing multiple spectral peaks that typically corresponded to three families of non-ribosomal lipopeptides, including iturins (1079.54 *m*/*z*, 1095.52 *m*/*z*, and 1093.884 *m*/*z*), surfactins (1044.961 *m*/*z*, 1030.959 *m*/*z*, and 1065.859 *m*/*z*), and fengycins (1058.60 *m*/*z* and 1074.97 *m*/*z*) (Figure 2). Furthermore, specific bands for genes involved in iturin- (*ItuB* and *ItuD*) and fengycin (*FenB*) biosynthesis, two well-known antifungal lipopeptide families, were PCR amplified using the selected primer pairs from the genomic DNA of *B. subtilis* DHA41 (Figure 3A). These data indicate that *B. subtilis* DHA41 possesses the inherent genetic potential for producing different extracellular lipopeptides. 

The emulsification index was analyzed to further confirm the presence of biosurfactants in the lipopeptide extract from *B. subtilis* DHA41. The lipopeptide extract showed emulsifying activity against two hydrophobic substrates, including mineral oil (31.25 ± 0.03%) and sunflower oil (51 ± 0.50%) (Figure 3B and Table 3). Interestingly, the lipopeptide extract did not show emulsifying activity against toluene (Figure 3B and Table 3). These results indicate that *B. subtilis* DHA41 is capable of producing extracellular biosurfactants in response to specific organic compounds. 

### 3.3. Antifungal Effect of Cell-Free Filtrate and Extracellular Lipopeptides of B. subtilis DHA41

**Figure 4:**
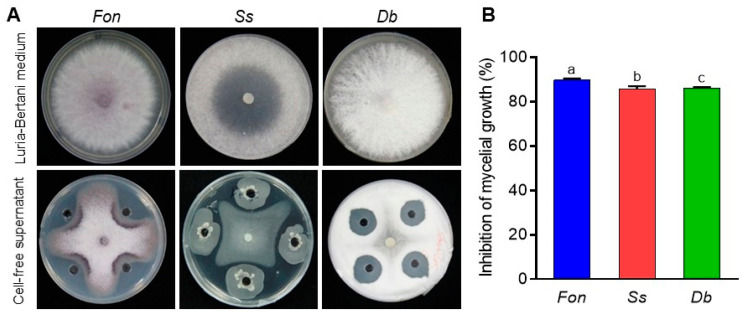
Antifungal activity of cell-free supernatant from *B. subtilis* DHA41 culture against *F. oxysporum* f. sp. *nevium* (*Fon*), *S. sclerotiourm* (*Ss*), and *D. byroniae* (*Db*). (**A**) Fungal colonies grown on potato dextrose agar supplemented with 60 µL of cell-free supernatant or sterile Luria–Bertani medium. (**B**) The inhibition rate of the cell-free supernatant against *Fon*, *Ss*, and *Db*. The experiment in (**A**) was independently performed three times with similar results. The data presented in (**B**) are the means ± standard deviation from three independent experiments. Different letters above the columns indicate a significant difference at 95% confidence level according to the one-way analysis of variance test.

The results of the antifungal activity of the cell-free supernatant of *B. subtilis* DHA41 against different pathogenic fungi showed significant inhibition of *Fon*, *Ss*, and *Db* mycelial growth (Figure 4A), with inhibition rates of 89.81%, 85.92%, and 86.06%, respectively, compared to the controls (Figure 4B). These results indicate the presence of potent antimicrobial compounds in the cell-free supernatant of *B. subtilis* DHA41 that effectively inhibit the mycelial growth of pathogenic fungi. 

**Figure 5:**
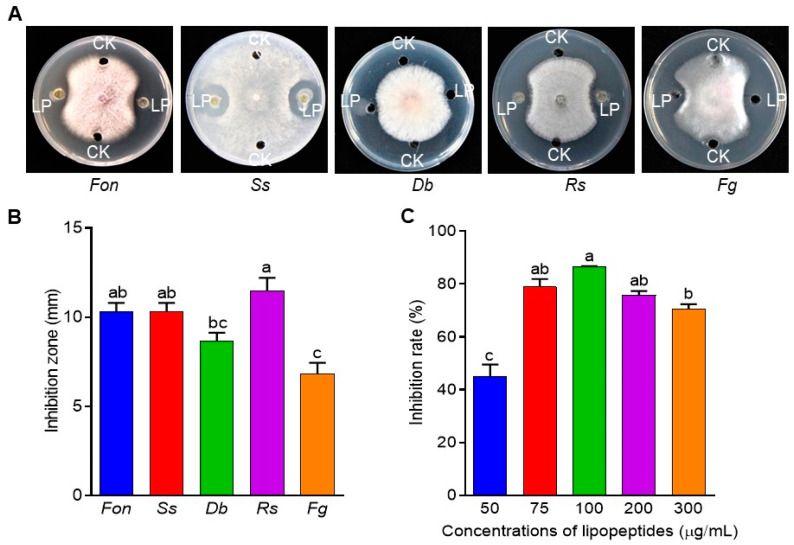
Antifungal activity of extracellular lipopeptide extract from *B. subtilis* DHA41 against *F. oxysporum* f. sp. *nevium* (*Fon*), *S. sclerotiourm* (*Ss*), *D. byroniae* (*Db*), *R. solani* (*Rs*), and *F. graminearum* (*Fg*). (**A**) Fungal colonies grown on potato dextrose agar supplemented with the extracellular lipopeptide extract (LP) or dimethyl sulfoxide (CK). (**B**) The inhibition zones of the LP against *Fon*, *Ss*, *Db*, *Rs*, and *Fg*. (**C**) The inhibition activity of different concentrations of the LP on *Fon* growth. The experiment in (**A**) was independently performed three times with similar results. The data presented in (**B**,**C**) are the means ± standard deviation from three independent experiments. Different letters above the columns indicate a significant difference at 95% confidence level according to the one-way analysis of variance test.

Next, inhibition zones were observed around the lipopeptide-supplemented wells for *Fon*, *Ss*, *Db*, *Rs*, and *Fg* colonies compared to the DMSO-supplemented control wells (Figure 5A). The largest inhibition zone was observed for *Rs* (11.50 ± 0.70 mm), followed by *Fon* (10.33 ± 0.47 mm), *Ss* (10.33 ± 0.47 mm), *Db* (8.66 ± 0.47 mm), and *Fg* (6.83 ± 0.62 mm) (Figure 5B). Furthermore, the MIC of the lipopeptide extract against *Fon* was determined. The extracellular lipopeptide extract at 100 µg/mL exhibited the highest inhibition rate (86.4%) against *Fon* (Figure 5C). However, *Fon* growth inhibition rate was decreased at higher concentrations of the lipopeptide extract (200 and 300 µg/mL) compared to 100 µg/mL (Figure 5C). The observed non-linear antifungal activity of the lipopeptide extract at higher concentrations might be attributed to the saturation effect and hormesis or a dose-dependent response. These results collectively indicate that the extracellular lipopeptide extract of *B. subtilis* DHA41 possesses significant antifungal activity against multiple phytopathogenic fungi, including *Fon*.

### 3.4. Extracellular Lipopeptides Disrupt Fon Cellular Integrity

**Figure 6:**
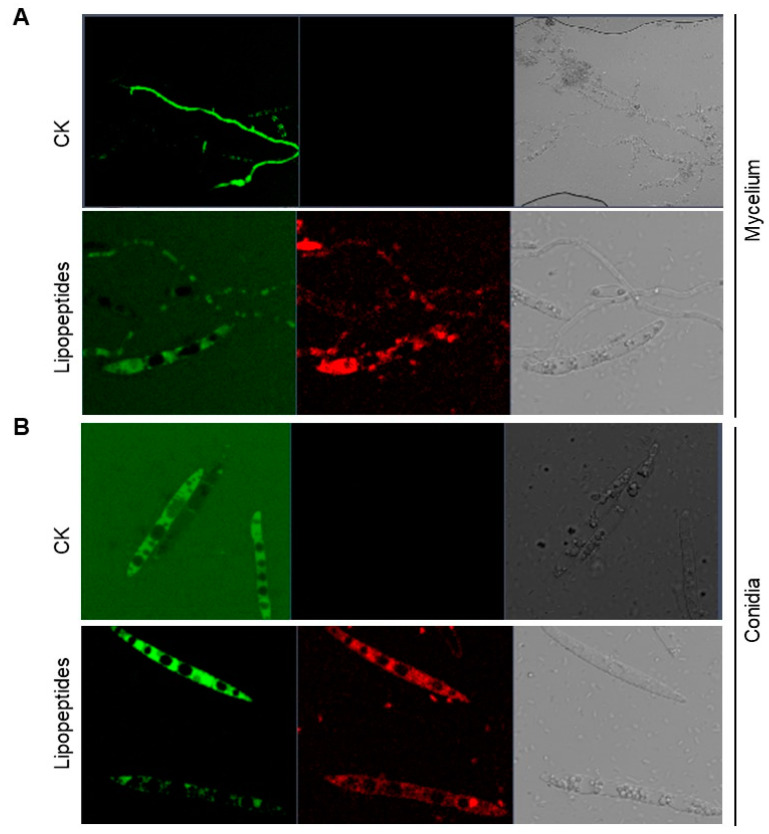
*B. subtilis* DHA41-produced lipopeptides affect cell viability in *F. oxysporum* f. sp. *niveum*. Lipopeptide extract- (30 µg/mL) or dimethyl sulfoxide-treated (CK) *Fon* mycelia (**A**) and conidia (**B**) were stained with fluorescein diacetate (FDA) and propidium iodide (PI) for 12 h, followed by detection of fluorescent signals using a Zeiss LSM 880 confocal laser microscope, with excitation at 488 nm for FDA and 561 nm for PI. Scale bar, 10 µm. The experiments were independently performed three times with similar results, and data from one representative experiment are shown.

The cell viability assays using FDA and PI staining [61,62] revealed that the untreated *Fon* mycelia and conidia exhibited normal morphology and intact structures, as evidenced from strong FDA-generated green fluorescent signals (Figure 6A,B). However, extracellular lipopeptide extract (30 µg/mL)-treated *Fon* mycelia and conidia showed PI-generated red fluorescence, revealing damaged morphology and collapsed structures (Figure 6). 

**Figure 7:**
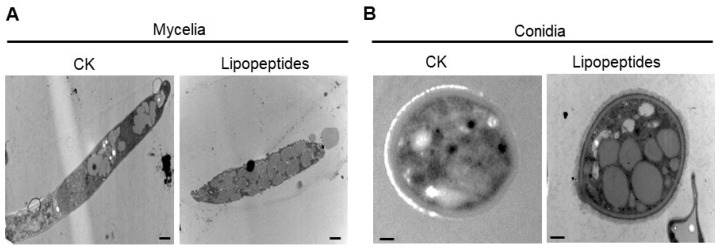
Ultrastructural changes in mycelia and conidia of *F. oxysporum* f. sp. *niveum* after treatment with *B. subtilis* DHA41-produced lipopeptide extract (30 µg/mL). Mycelia (**A**) and conidia (**B**) of *Fon* were treated with lipopeptide extract or dimethyl sulfoxide (CK) for 12 h and then examined under a transmission electron microscope (TEM). Scale bars, representing 2 μm in (**A**) and 1 μm in (**B**), respectively, are shown at the bottom of the TEM images. The experiments were independently performed three times with similar results, and data from one representative experiment are shown.

Further, the ultrastructure studies demonstrated that the untreated mycelia and conidia showed intact cellular morphology and structures, such as cellular membranes and cytoplasm, while mycelia and conidia treated with the lipopeptide extract displayed abnormal morphology and structure, as revealed by shrinking of the cytoplasm, plasma membrane damage, and cell wall disintegration (Figure 7). These data indicate that the extracellular lipopeptide extract of *B. amyloliquefaciens* DHA41 can disrupt *Fon* integrity, leading to cellular damage and decreased viability.

### 3.5. Antifungal Effect of the VOCs of B. subtilis DHA41

**Figure 8:**
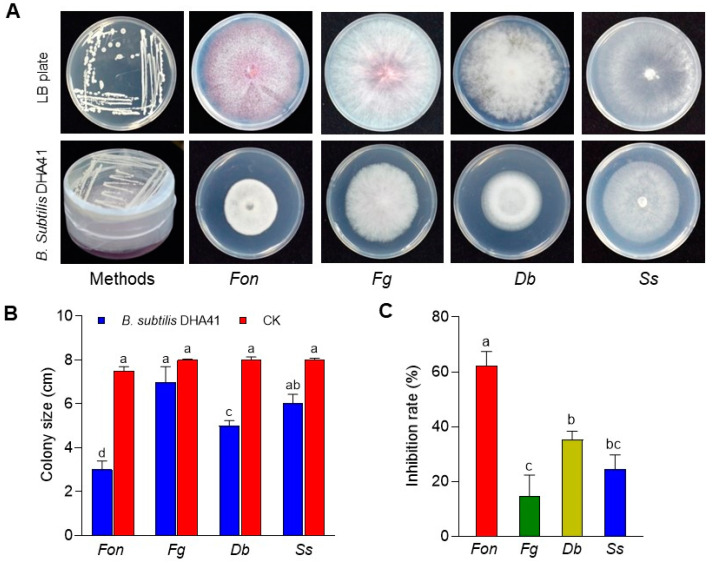
Antifungal activity of *B. subtilis* DHA41-emitted volatile organic compounds against *F. oxysporum* f. sp. *nevium* (*Fon*), *F. graminearum* (*Fg*), *D. byroniae* (*Db*), and *S. sclerotiourm* (*Ss*). (**A**) Fungal colonies grown with or without *B. subtilis* DHA41 in the two-sealed-base-plate experiments. (**B**) Colony sizes of the tested fungi grown with *B. subtilis* DHA41. (**C**) Inhibition rates of colony growth of the tested fungi grown with *B. subtilis* DHA41. The experiment in (**A**) was independently performed three times with similar results. The data presented in (**B**,**C**) are the means ± standard deviation from three independent experiments. Different letters above the columns indicate a significant difference at 95% confidence level according to the one-way analysis of variance test.

*Bacillus* spp. are well-known for producing diverse antifungal VOCs [32,35]. In this study, the results revealed that *Fon, Ss, Db,* and *Fg* growth was significantly reduced when co-cultivated with *B. subtilis* DHA41, compared to the controls without strain DHA41 (Figure 8A). Among the tested fungi co-cultured with *B. subtilis* DHA41, *Fon* displayed the smallest colony growth (3.0 ± 0.4 cm), followed by *Db* (5.01 ± 0.23 cm), *Ss* (6.03 ± 0.41 cm), and *Fg* (7.0 ± 0.7 cm), leading to growth inhibition rates of 62.3 ± 5.1%, 35.4 ± 2.9%, 24.6 ± 5.1%, and 14.6 ± 7.8%, respectively (Figure 8B,C). These results indicate that *B. subtilis* DHA41 emits antifungal VOCs, inhibiting the radial growth of various soil-borne pathogenic fungi, such as *Fon*, *Fg*, *Db*, and *Ss*.

## 4. Discussion

Biological control has become crucial in integrated crop disease management systems [17,63,64,65]. Recent studies have shown that manipulating the soil microbiomes with plant-beneficial bacterial species suppressed Fusarium wilt in watermelon [66]. Several beneficial bacterial strains, such as *Paenibacillus polymyxa*, *Pseudomonas fluorescens*, *Streptomyces goshikiensis*, and *Bacillus* spp., have demonstrated efficacy in controlling Fusarium wilt in watermelon [67,68,69]. Particularly, strains of *B. subtilis*, *B. amyloliquefaciens*, *B. velezensis*, and *B. methylotrophicus* have showed significant antifungal activity against *Fon* and effectively suppressed Fusarium wilt in watermelon [19,36,38,70,71,72,73,74]. However, the underlying mechanisms of watermelon Fusarium wilt suppression by these beneficial bacterial strains remain unclear. We previously characterized six antagonistic bacterial strains, including DHA41, against various soil-borne pathogenic fungi, including *Fon*, which successfully suppressed watermelon Fusarium wilt in greenhouse experiments [16]. The present study investigated the biocontrol mechanism of DHA41, which synthesized antifungal bioactive compounds, including extracellular lipopeptides and VOCs, showing significant inhibitory activities against *Fon* and other tested soil-borne fungal pathogens. 

In our previous study, DHA41 was shown to be a gram-positive rod-shaped bacterial strain capable of producing catalase, protease, cellulase, ammonium, indole-3-acetic acid, siderophore, and solubilizing inorganic phosphate [16]. Phylogenetic analysis of *16S rRNA* and fatty acid profiling confirmed that strain DHA41 belongs to *B. subtilis* (Figure 1 and Table 2). Notably, *B. subtilis* DHA41 exhibited high levels of 16:0, 15:0 iso, and 15:0 anteso fatty acids, which aligns with the typical fatty acid composition commonly found in *Bacillus* spp. [59,60]. 

It has previously been discovered that the non-ribosomally synthesized extracellular lipopeptides are associated with the antimicrobial activity and biocontrol of plant diseases by *Bacillus* spp. [29,31]. Generally, *Bacillus* spp. produce three families of lipopeptides, including surfactins, fengycins, and iturins [29]. In the present study, MALDI-TOF-MS analysis identified three isoforms of iturins, three isoforms of surfactins, and two isoforms of fengycins (Figure 2), indicating the genetic diversity of *B. subtilis* DHA41 to produce extracellular lipopeptides. This result is further confirmed by detecting *ItuB*, *ItuD*, and *FenB* genes in *B. subtilis* DHA41 (Figure 3A). Comparative genomic studies have shown that *B. subtilis* strains harbor 11 putative large biosynthetic gene clusters, some of which are responsible for lipopeptide production [75]. Recent studies have also highlighted variations in the production of non-ribosomally synthesized lipopeptides among different *B. subtilis* strains isolated from the same soil sample [76]. For example, analysis of 330 biosynthetic clusters from *B. subtilis* and their lipopeptides revealed a species-specific pattern of lipopeptide production [77]. Furthermore, *B. subtilis* DHA41 exhibited significant emulsification activity against some organic oils, such as mineral oil and sunflower oil (Figure 3B and Table 2), which coincides with previous studies where lipopeptide biosurfactants from *Bacillus thuringiensis* pak2310 showed emulsification and antifungal activity against *F. oxysporum* [78]. The emulsification activity is known to contribute to adhesion, bioavailability, desorption, and antimicrobial activity in natural environments [79]. Collectively, the diverse extracellular lipopeptides and emulsification activity play an important role in the antifungal activity and disease-suppressing ability of *B. subtilis* DHA41.

The cell-free supernatant and extracellular lipopeptide extract of *B. subtilis* DHA41 exhibited significant broad-spectrum antifungal activity against *Fon*, *Db*, *Ss*, *Rs*, and *Fg* (Figure 4A,B). This finding is consistent with previous observations showing that lipopeptide extracts from *B. subtilis* SCB-1 and *B. amyloliquefaciens* CNU114001 showed antifungal activity against diverse fungal pathogens, including *F. oxysporum* and *Ss* [80,81]. The lipopeptide extract from *B. subtilis* DHA41 had a notable impact on the viability of mycelia and conidia of *Fon* (Figure 6), leading to disruptions in cellular structure and integrity (Figure 7). These observations align with previous results demonstrating that lipopeptides from *B. velezensis* and *B. amyloliquefaciens* caused morphological changes in *Fon*, such as cytoplasmic shrinkage, aggregation of organelles, and damage to plasma membranes and cell walls [36,38,53,71]. Furthermore, the *Bacillus* spp.-produced lipopeptides enter *Fon* cells through endocytosis [82], subsequently targeting intracellular molecules and triggering metabolic alterations, thus exerting the antifungal effects against *Fon* [72,83].

In addition to extracellular lipopeptides, antagonistic bacteria also emit a wide range of VOCs [32,35]. These VOCs are low molecular weight compounds that readily emit under normal environmental conditions and exhibit significant antimicrobial activity [33]. In this study, co-incubation of *Fon*, *Db*, *Ss*, and *Fg* with *B. subtilis* DHA41 significantly inhibited the mycelial growth of fungal pathogens, implying the production of VOCs by *B. subtilis* DHA41 (Figure 8). Notably, *B. subtilis* DHA41-emitted VOCs exhibited varying levels of inhibition against the tested fungal pathogens. *B. subtilis*, *B. amyloliquefaciens*, and *B. mycoides* have been reported to release antifungal VOCs, inhibiting the mycelial growth and spore germination rate of *Fon*, *F. oxysporum* f. sp. *lycopersici*, *F. oxysporum* f. sp. *cubense*, *F. oxysporum* f. sp. *radicis-lycopersici*, and *Ss* [40,41,42,43,84]. For example, *B. amyloliquefaciens* L3-produced VOCs, including 2-heptanone, 2-ethyl-1-hexano, and 2-nonanone, completely inhibited *Fon* mycelial growth [40]. VOCs of antagonistic bacteria have been shown to improve plant growth and trigger induced systemic resistance in plants [33]; for example, albuterol and 1,3-propanediole, two volatile organic compounds produced by *B. subtilis* SYST2, and acetoin and 2,3-butanediol, produced by *B. amyloliquefaciens* L3, promoted the growth of tomato and Arabidopsis plants [73,85]. However, the chemical nature of the *B. subtilis* DHA41-produced VOCs and their involvement in promoting plant growth and suppressing Fusarium wilt in watermelon [16] need further investigation.

## 5. Conclusions

In this study, we found that the antagonistic bacterium *B. subtilis* DHA41 produces three families of extracellular lipopeptides, including iturins, surfactins, and fengycins, which exhibited significant antifungal activity against five soil-borne phytopathogenic fungi, including *Fon*, *Db*, *Ss*, *Rs*, and *Fg*. The extracellular lipopeptide extract of *B. subtilis* DHA41 effectively inhibited the mycelial growth and spore germination of *Fon* by disrupting cellular structure and integrity. Furthermore, *B. subtilis* DHA41 emitted VOCs that displayed inhibitory effects on the mycelial growth of *Fon*, *Db*, *Ss*, and *Fg*. Our findings highlight the biocontrol capacity of *B. subtilis* DHA41 in combating Fusarium wilt in watermelon through the production of diverse extracellular lipopeptides and VOCs. The ability of *B. subtilis* DHA41 to promote plant growth and suppress Fusarium wilt in watermelon, along with the antifungal activity of its active secondary metabolites, suggests the possibility of developing *B. subtilis* DHA41-based biopesticides for the protection of important crops against soil-borne diseases under field conditions.

## Figures and Tables

**Table 1:** The primers used in this study.

Genes	Primers	Sequences (5′-3′)	Size (bp)	References
*16S rRNA*	27F	AGAGTTTGATCATGGCTCAG	1081	[48]
	1479R	TACGGTTACCTTGTTACGACTT		
*ItuB*	BamB2F	CGACATACAGTTCTCCCCGGT	461	[49]
	BamB2R	AAGAAGGCGTTTTTCAAGCA		
*ItuD*	ItuD1F	GACGGTAGATTCGCTGCTGT	583	[49]
	ItuD1R	TGATGCGATCTCCTTGGATG		
*FenD*	FNDF2	CTGGGAGGTCAGCCGGTCTG	216	[49]
	FNDR2	GTGGTCGCCGGTTCACAAAT		

**Table 2:** The composition of fatty acids in *B. subtilis* DHA41.

Fatty Acids (FA)	Content (%)
Saturated straight chain FA	
12:0	--
14:0	2.67
16:0	15.42
18:0	0.54
Saturated terminally branched FA	
13:0 iso	0.51
14:0 iso	1.55
15:0 iso	24.55
16:0 iso	1.79
17:0 iso	6.65
15:0 anteso	34.10
17:0 anteso	5.20
Monounsaturated FA	
16:1 w11c	4.06
17:1ω10c iso	1.46

**Table 3:** Emulsification index of lipopeptide extract from *B. subtilis* DHA41 on different organic oil substrates.

Mineral Oil	Sunflower Oil	Toluene
51 ± 0.50	31.25 ± 0.03	nd

nd, not detectable.

## Data Availability

All the data are present inside the manuscript file.
